# Cannabis use, sedentary behavior, and physical activity in a nationally representative sample of US adults

**DOI:** 10.1186/s12954-021-00496-2

**Published:** 2021-04-29

**Authors:** Lydia Q. Ong, John Bellettiere, Citlali Alvarado, Paul Chavez, Vincent Berardi

**Affiliations:** 1 Department of Psychology, University of British Columbia, 2136 West Mall, Vancouver, BC V6T 1Z4 USA; 2 Herbert Wertheim School of Public Health and Longevity Science, University of California San Diego, 9500 Gillman Drive, San Diego, CA 92093 USA; 3 Graduate School of Public Health, San Diego State University, 5500 Campanile Drive, San Diego, CA 92182 USA; 4 Department of Psychology, Chapman University, 1 University Drive, Orange, CA 92866 USA

**Keywords:** Cannabis, Sedentary behavior, Physical activity, Accelerometry, Behavioral epidemiology

## Abstract

**Background:**

Prior research examining the relationship between cannabis use, sedentary behavior, and physical activity has generated conflicting findings, potentially due to biases in the self-reported measures used to assess physical activity. This study aimed to more precisely explore the relationship between cannabis use and sedentary behavior/physical activity using objective measures.

**Methods:**

Data were obtained from the 2005–2006 National Health and Nutrition Examination Survey. A total of 2,092 participants (ages 20–59; 48.8% female) had accelerometer-measured sedentary behavior, light physical activity, and moderate-to-vigorous physical activity. Participants were classified as light, moderate, frequent, or non-current cannabis users depending on how often they used cannabis in the previous 30 days. Multivariable linear regression estimated minutes in sedentary behavior/physical activity by cannabis use status. Logistic regression modeled self-reported moderate-to-vigorous physical activity in relation to current cannabis use.

**Results:**

Fully adjusted regression models indicated that current cannabis users’ accelerometer-measured sedentary behavior did not significantly differ from non-current users. Frequent cannabis users engaged in more physical activity than non-current users. Light cannabis users had greater odds of self-reporting physical activity compared to non-current users.

**Conclusions:**

This study is the first to evaluate the relationship between cannabis use and accelerometer-measured sedentary behavior and physical activity. Such objective measures should be used in other cohorts to replicate our findings that cannabis use is associated with greater physical activity and not associated with sedentary behavior in order to fully assess the potential public health impact of increases in cannabis use.

## Background

Regular physical activity (PA) is associated with a host of health benefits, such as reduced risks for cardiovascular disease, diabetes, and all-cause mortality, while sedentary behavior (SB; e.g., sitting while watching television [1]) is linked with increased health risks [2–4]. However, less than one quarter of US adults aged 18–64 meet federally recommended PA guidelines [5]. This insufficiency likely plays a role in the high prevalence of several cardiometabolic conditions, including metabolic syndrome, obesity, cardiovascular disease, and diabetes that the USA currently experiences [6–9]. This trend in morbidity has coincided with, and possibly been affected by, an increase in cannabis use and a decrease in perceived risk over the past two decades in the USA [10]. While research has shown that cannabis users have lower odds of metabolic syndrome, obesity, and other cardiometabolic disease risk factors [11–14], these findings have typically not considered lifestyle factors, which play a significant role in the prevention and management of chronic conditions [15–17]. One important lifestyle consideration is physical behavior (which encompasses both SB and PA), whose relationship with cannabis use is not well understood.

Research on the association between cannabis use and physical behavior in North America is scant, with previous studies producing inconsistent findings. In the USA, more than 80% of participants in states with full legal access to cannabis reported concurrent cannabis use with exercise [18]. Among a nationally representative sample, an examination of eight years of data from the National Health and Nutrition Examination Survey (NHANES) suggested that current cannabis users had lower odds of engaging in self-reported PA compared to non-current users [19], whereas a different study using ten years of NHANES data found that any lifetime cannabis use was associated with higher odds of being physically active [20]. A study utilizing NHANES data found that females with any lifetime cannabis use had greater self-reported sedentary time compared to females who had never used cannabis [20]. Finally, a population-based survey of Canadians found that, compared to nonusers, cannabis users had greater odds of spending over 35 h per week in sedentary behavior [21].

To date, studies examining the relationship between cannabis use and physical behavior have relied on self-report measures via questionnaires, which are subject to recall [22] and social desirability [23] biases that have the potential to generate inaccuracies. Specific to PA, it may be easier to report on discrete events (e.g., “Have you gone on a run in the past 7 days?”) versus recalling the amount of time engaged in various intensities of PA. Moreover, regular cannabis use is known to impair memory recall [24, 25], possibly reducing accurate self-reported measures of physical behavior among cannabis users. As an alternative, accelerometers (e.g., Fitbit) have become a common tool for *objectively* assessing physical behavior. A systematic review found weak-to-moderate correlations between accelerometry measures and PA questionnaires (0.08 $\le$ *r* $\le$ 0.58; [26]). Another study displayed even weaker correlations between accelerometry measures and light PA (0.01 $\le$ *r* $\le$ 0.06) and moderate correlations for accelerometry and SB (0.28 $\le$ *r* $\le$ 0.31; [27]). These inconsistencies raise concerns over the accuracy of using questionnaires to assess the relationship between cannabis use and physical behavior.

The current study aimed to account for potential shortcomings in self-report assessments within cannabis and physical behavior studies by incorporating objective measures of SB, light PA, and MVPA into the analyses. We analyzed NHANES accelerometer and questionnaire data to investigate differential levels of SB and PA among current cannabis users and non-current users. Our aim was to clarify the relationship between cannabis use and physical behavior to inform the evaluation of changes in physical behavior patterns as a potential public health impact of cannabis use.

## Methods

### Sample description

Data were obtained from NHANES, a program of studies by the National Center for Health Statistics which assesses the health and nutritional status of the US population through questionnaires, interviews, physical examinations, and laboratory tests [28]. NHANES uses a stratified, multistage probability sampling design to attain a nationally representative sample of the non-institutionalized US population. The sampling procedure begins with selecting primary sampling units (counties) which are then divided into segments (city blocks). A sample of households within each segment is randomly drawn, and individuals within those households are randomly selected. Only the 2005–2006 NHANES cycle has concurrent accelerometer and cannabis use measures, so analyses were restricted to this time period. The sample was limited to participants aged 20–59 years (n = 3409), since only respondents in this age range were given drug and alcohol use questionnaires. Participants were excluded if they did not have one or more adherent accelerometer wear days, defined as having at least 10 h of wear time (n = 652; [29]); did not complete the Drug Use Questionnaire (n = 124); had incomplete cannabis use questions (n = 303); reported concurrent use of heroin, cocaine, or methamphetamines (n = 30); or self-reported or tested positive for pregnancy via urine test (n = 208). The final analytic sample contained 2,092 participants.

### Measures of cannabis use

Using responses from the Drug Use Questionnaire [30], cannabis use was determined by responses to the following questions: “Have you ever, even once, used marijuana or hashish?” (yes, no). Individuals who responded “yes” were directed to the following question: “During the past 30 days, on how many days did you use marijuana or hashish?”. Current cannabis users were defined as those who used cannabis ≥ 1 day in the past 30 days [31]. We further divided current cannabis users by frequency of use, which may have differing impacts on physical behavior. As done in previous research [19], we classified current cannabis users into light users (< 10 days in the past 30 days), moderate users (10–20 days in the past 30 days), and frequent users (> 20 days in the past 30 days). All other participants were classified as non-current users.

### Accelerometer measures of physical activity and sedentary behavior

Participants were requested to wear an Actigraph 7164 over their right hip for 7 days and to remove the device only for showering, swimming, and when in bed. The accelerometer data were processed via a computer script released by the National Cancer Institute, the full details of which are published elsewhere [32]. Briefly, using the most common protocol for adults, accelerometer non-wear was characterized by 60 consecutive minutes with zero movement allowing for up to 2 min of movement below 50 counts per minute (cpm; [29]). As is commonly defined, an adherent day was designated as having at least 10 h of wear time [29]. Common acceleration cutpoints were then used to categorize different behaviors: minutes with less than 100 cpm were classified as sedentary, minutes between 100 and 1951 cpm were classified as light PA, and minutes above 1951 cpm were considered MVPA [33, 34]. The physical behavior metrics used in all analyses were computed as the average number of minutes in sedentary time, light PA, and MVPA over all adherent days.

### Measures of self-reported physical activity

Participants self-reported their levels of PA through the physical activity questionnaire [30]. Engagement in MVPA was determined by the following questions: “Over the past 30 days, did you do moderate activities for at least 10 min that cause only light sweating or a slight to moderate increase in breathing or heart rate?” (yes, no); “Over the past 30 days, did you do any vigorous activities for at least 10 min that caused heavy sweating, or large increases in breathing or heart rate?” (yes, no). Responses to these questions were combined into one binary yes/no variable; if a respondent replied “yes” to one or both questions, they were categorized as having self-reported MVPA engagement. This method of measurement was chosen to replicate a previous study that used NHANES data to examine the cannabis-PA relationship [19]. Self-reported light PA and SB were not assessed due to lack of appropriately corresponding questions in 2005–2006 NHANES.

### Covariates

Sociodemographic covariates included age, sex, race/ethnicity, income-to-poverty ratio, and education. Health-related covariates included body mass index (BMI), cigarette smoking status, and alcohol use. All covariates were obtained through self-report with the exception of BMI, which was measured by trained examiners. Age was reported in years, and sex was indicated as male or female. Race/ethnicity was categorized as non-Hispanic White, non-Hispanic Black, Hispanic (inclusive of Mexican American and Other Hispanic), or Other (inclusive of Asian and multi-racial); non-Hispanic White was the reference level. Income-to-poverty ratio was calculated by dividing family income by the poverty threshold. Education was indicated as one of five categories: less than 9th grade, 9th–11th grade, high school graduate/GED, some college/AA degree, or college graduate and above (less than 9th grade was the reference level). Body mass index was calculated based on height and weight measurements. Cigarette smoking status was determined by the following questions: “Have you smoked at least 100 cigarettes in your entire life?” (yes, no) and “Do you now smoke cigarettes?” (every day, some days, not at all). Current cigarette smokers were classified as those who answered “yes” to smoking at least 100 cigarettes in their lifetime and now smoke “every day” or “some days.” Alcohol use was defined by the average number of days per week participants drank and was assessed using the following questions: “In your entire life, have you had at least 12 drinks of any type of alcoholic beverage?” (yes, no); “In the past 12 months, how often did you drink any type of alcoholic beverage? How many days per week, per month, or per year did you drink?” (responses recorded from 0–365). Responses were converted to days per week as applicable. Participants who have not had at least 12 alcoholic drinks in their lifetime or responded with “0” to frequency of drinking in the past 12 months were recorded as drinking an average of 0 days per week.

### Statistical analyses

All statistical analyses were performed in R version 4.0.4 [35] using the survey package [36], which was use to account for NHANES sample weights, strata, and primary sampling units to make results more generalizable to the US population. To determine statistical significance, α was set to 0.05.

Means and frequencies of covariates were calculated for the total sample and separately by cannabis use categories. Differences between these groups were tested using *t* tests for continuous variables and χ^2^ tests for categorical variables, both of which accounted for the survey design.

To account for differences in physical behavior measurements resulting from differences in the duration of wear time, SB, light PA, and MVPA were adjusted for accelerometer wear time via the commonly used [32, 37] residuals method [38]. Linear regression models assessed associations of cannabis use with SB, light PA, and MVPA as separate outcome variables using the following successively adjusted regression models: Model 1 (unadjusted), Model 2 (adjusted for age and sex), and Model 3 (adjusted for age, sex, ethnicity, income-to-poverty ratio, education, body mass index, cigarette smoking status, and alcohol use). Models 1 and 2 contained 2,092 observations, while Model 3 contained 2,022 observations due to missing income-to-poverty ratio, BMI, or alcohol use data. To facilitate interpretation, regression results are reported as marginal means [36].

To assess potential effect modification, interaction terms for cannabis use and age, sex, and cigarette smoking status were separately added to Model 3. Previous research has documented age and sex differences in SB time [39] and associations between cigarette smoking and cannabis dependence [40]. Marginal means of SB, light PA, and MVPA were estimated for current and non-current cannabis users and differences were computed and reported separately for each level of the potential modifiers in Model 3. Age was dichotomized into younger or older than the median sample age (40 years) for the reporting of marginal means but was treated as continuous in the models. Statistical significance of the between-level differences was obtained from the *p*-value of the interaction coefficients in the regression model.

To replicate a previous study [19], logistic regression models were calculated with self-reported MVPA as a binary outcome. Three logistic models were created using the same covariates and sample sizes as the linear accelerometry models (without interaction terms).

Two sets of sensitivity analyses were conducted. First, the non-interaction regression models were re-analyzed for a subsample of participants that had a minimum of four adherent accelerometer wear days (*n* = 1618), an eligibility criterion thought to produce physical behavior estimates that more accurately represent usual weekly behavior patterns. Second, analyses were re-analyzed on a subsample of participants without the following medical conditions: diabetes, arthritis, heart failure, coronary heart disease, and cancer (*n* = 1551).

## Results

### Descriptive statistics

Current cannabis users differed significantly from non-current users on all included sociodemographic and health-related covariates (Table 1). Current cannabis users were younger (mean age 34.2 vs. 41.1 years), more likely to be White (54.2% vs. 46.4%), male (71.5% vs. 48.5%), have higher rates of some college/AA degree (39.4% vs. 33.4%), have lower BMI (mean 26.5 vs. 28), and have a lower income-to-poverty ratio (mean 2.8 vs. 3.3). Approximately half of cannabis users were current cigarette smokers (51%), and they drank alcoholic beverages an average of 2 days per week.

### Sedentary behavior, light PA, and MVPA

There were no significant differences between non-current cannabis users and light, moderate, or frequent cannabis users in minutes per day spent in SB. However, current users differed from non-current users in time engaged in PA. After controlling for all covariates, frequent cannabis users engaged in significantly greater amounts of light PA and MVPA compared to non-current users (light PA: 391.9 min/day (95% CI [366.7, 417.1]) vs. 350.2 min/day (95% CI [340.7, 359.6]), *p* = 0.01; MVPA: 37.1 min/day (95% CI [29.0, 45.3]) vs. 27.2 min/day (95% CI [25.9, 28.6]), *p* = 0.03; Table 2). In the unadjusted model, moderate cannabis use predicted more minutes spent in MVPA compared to non-current use (34.3 min/day (95% CI [26.1–42.5]) vs. 26.5 min/day (95% CI [25.0–28.0]), *p* = 0.03), but this association was not significant upon controlling for all covariates. Light cannabis users did not significantly differ from non-current users in time engaged in PA.

**Table 1:** Descriptive statistics of adults with completed drug questionnaire and accelerometer use, national health and nutrition examination survey 2005–2006.

	Overall sample *N* = 2,092	Current users *n* = 249	Non-current users *n* = 1,843	*p*
Age, mean (SD)	40.3 (11.1)	34.2 (10.8)	41.1 (10.9)	** < 0.001**
Sex, *n* (%)				** < 0.001**
Male	1072 (51.2)	178 (71.5)	894 (48.5)	
Female	1020 (48.8)	71 (28.5)	949 (51.5)	
Race/Ethnicity, *n* (%)				** < 0.01**
Non-Hispanic White	990 (47.3)	135 (54.2)	855 (46.4)	
Hispanic	511 (24.4)	33 (13.3)	478 (25.9)	
Non-Hispanic Black	501 (24)	71 (28.5)	430 (23.3)	
Other/Multi-Racial	90 (4.3)	10 (4.0)	80 (4.3)	
Income-to-poverty ratio, mean (SD)^a^	3.3 (1.6)	2.8 (1.6)	3.3 (1.6)	** < 0.002**
Education level, *n* (%)				** < 0.002**
< 9th Grade	161 (7.7)	8 (3.2)	153 (8.3)	
9th–11th Grade	281 (13.4)	52 (20.9)	229 (12.4)	
High school Graduate/GED	480 (22.9)	65 (26.1)	415 (22.5)	
Some college/AA degree	714 (34.1)	98 (39.4)	616 (33.4)	
College graduate or above	456 (21.8)	26 (10.4)	430 (22.3)	
Body mass index, mean (SD) Current cigarette smoker, *n* (%)	28.7 (7) 449 (21.5)	26.5 (5.3) 126 (51)	28 (7.1) 323 (17.5)	** < 0.001** ** < 0.002**
Alcohol use, mean (SD)^a^	1.1 (1.8)	2.1 (2.2)	1.0 (1.7)	** < 0.001**

SD = Standard deviation; GED = General education development or high school equivalency; AA = associates of arts. Bolded text represents statistically significant effects.

### Interaction effects

Age interacted with moderate cannabis use to predict MVPA, such that moderate cannabis users above age 40 engaged in 16 additional minutes (95% CI [5.5, 26.7]) of MVPA per day than their nonuser counterparts; in contrast, moderate cannabis users below age 40 engaged in 3.5 fewer minutes (95% CI [(− 7.5, 0.4]) of MVPA per day than their nonuser counterparts (*p* = 0.02). Additionally, cigarette smoking status moderated the association between light cannabis use and MVPA. Only light cannabis users who did not currently smoke cigarettes engaged in more minutes of MVPA compared to non-current cannabis users (1.8 min/day (95% CI [(− 1.4, 4.9]) vs. − 6.2 min/day (95% CI [−9.2, −3.2]), *p* = 0.02). There were no observed sex differences in the associations between cannabis use and physical behavior (Table 3).

**Table 2:** Estimated time spent engaged in physical behavior by cannabis use category

	Non-current users *n* = 1843	Light users *n* = 144	*p*	Moderate users *n* = 50	*p*	Frequent users *n* = 55	*p*
	Mean (min/day) (95% CI)	Mean (min/day) (95% CI)		Mean (min/day) (95% CI)		Mean (min/day) (95% CI)	
Sedentary behavior							
Model 1	482.9 (475.1–490.7)	465.2 (438.6–491.8)	0.22	463.9 (412.8–515.0)	0.48	437.4 (382.6–492.2)	0.13
Model 2	482.3 (463.2–501.4)	470.4 (444.8–496.0)	0.39	463.9 (412.8–515.0)	0.54	442.5 (387.8–497.2)	0.18
Model 3	481.2 (471.8, 490.7)	478.7 (450.5, 506.8)	0.86	479.4 (431.0, 527.7)	0.94	458.7 (397.1, 520.3)	0.50
Light physical activity							
Model 1	348.3 (339.5–357.1)	357.2 (331.0–383.4)	0.52	374.9 (310.9–438.9)	0.43	407.0 (380.5–433.5)	** < 0.001**
Model 2	348.2 (324.5–371.9)	358.2 (332.2–384.2)	0.47	374.4 (313.3–435.5)	0.42	407.6 (380.9–434.3)	**0.001**
Model 3	350.2 (340.7, 359.6)	347.6 (326.1, 369.1)	0.85	367.6 (323.5, 411.8)	0.44	391.9 (366.7, 417.1)	**0.01**
Moderate-to-vigorous physical activity							
Model 1	26.5 (25.0–28.0)	29.1 (24.0–34.2)	0.18	34.3 (26.1–42.5)	**0.03**	40.6 (32.4–48.8)	**0.006**
Model 2	27.1 (23.0–31.2)	25.9 (22.1–29.7)	0.54	28.3 (22.1–34.5)	0.70	35.1 (27.5–42.7)	0.07
Model 3	27.2 (25.9, 28.6)	25.4 (21.4, 29.4)	0.35	30.3 (24.0, 36.6)	0.36	37.1 (29.0, 45.3)	**0.03**

Non-current users were the reference group. All estimates are weighted due to the survey design. Model 1: unadjusted model (*N* = 2,092); Model 2: adjusted for age and sex (*N* = 2,092); Model 3: adjusted for age, sex, race/ethnicity, education, income-to-poverty ratio, body mass index, cigarette smoker status, and alcohol use (*N* = 2,022). CI = Confidence interval. Bolded text represents statistically significant effects

### Self-reported MVPA

A majority of current and non-current cannabis users self-reported engagement in MVPA in the past 30 days (light users: 79.6%; moderate users: 65.7%; frequent users: 61.7%; non-current users: 71.8%). Results from the logistic regression models indicated that light cannabis users had 1.09 times greater odds of self-reported MVPA (95% CI [1.03, 1.15], *p* = 0.01) compared to non-current users, whereas moderate and frequent users did not significantly differ from non-current users in their odds of self-reporting MVPA (Table 4).

**Table 3:** Differences between current cannabis users versus non-current users in time spent in physical behavior stratified by age, sex, and current cigarette smoking status

	Age			Sex			Current cigarette smoker		
	$\le$ 40	> 40	*p*	Male	Female	*p*	Yes	No	*p*
	Difference in Mean (min/day) (95% CI)			Difference in Mean (min/day) (95% CI)			Difference in Mean (min/day) (95% CI)		
Light users (*n* = 142)									
Sedentary behavior	15.7 (− 8.8, 40.1)	− 32.9 (− 61.5, − 4.3)	0.21	8.8 (− 11.4, 28.9)	− 17.9 (− 43.7, 7.9)	0.31	10.5 (− 14.7, 35.7)	− 13.6 (− 33.7, 6.5)	0.35
Light physical activity	− 19.6 (− 44.2, 5.3)	18.1 (3.4, 32.7)	0.20	− 2.9 (− 21.9, 16.1)	− 1.1 (− 28.1, 25.9)	0.95	− 15.9 (− 49.1, 17.4)	8.5 (− 7.9, 24.8)	0.45
Moderate-to-vigorous physical activity	− 2.0 (− 6.2, 2.3)	0.3 (− 4.2, 4.8)	0.39	− 4.0 (− 8.3, 0.3)	1.2 (− 3.4, 5.7)	0.30	− 6.2 (− 9.2, − 3.2)	1.8 (− 1.4, 4.9)	**0.02**
Moderate users (*n* = 47)									
Sedentary behavior	23.2 (− 28.6, 75.1)	− 32.9 (− 94.1, − 12.6)	0.17	15.5 (− 88.1, 70.2)	9.1 (− 73.2, 91.3)	0.77	− 18.1 (− 31.5, − 4.7)	19.2 (− 52.0, 90.3)	0.42
Light physical activity	− 1.9 (− 47.2, 43.4)	49.2 (9.0, 89.4)	0.18	31.3 (− 3.0, 65.5)	− 37.7 (− 93.8, 18.4)	0.07	26.3 (− 2.9, 55.4)	5.6 (− 56.1, 67.2)	0.61
Moderate-to-vigorous physical activity	− 3.5 (− 7.5, 0.4)	16.1 (5.5, 26.7)	**0.02**	4.1 (− 0.5, 8.6)	− 2.3 (− 10.3, 5.7)	0.20	8.4 (2.8, 14.1)	− 3.9 (− 9.5, 1.7)	0.07
Frequent users (*n* = 54)									
Sedentary behavior	4.0 (− 62.1, 70.1)	− 68.3 (− 143.8, 7.1)	0.59	− 1.9 (− 56.6, 52.8)	− 78.9 (− 175.0, 17.2)	0.14	− 24.7 (− 75.3, 25.9)	− 12.2 (− 96.3, 72.0)	0.85
Light physical activity	39.6 (23.3, 55.9)	39.2 (1.6, 76.7)	0.98	45.2 (23.8, 66.5)	33.4 (− 5.8, 72.6)	0.71	45.6 (31.9, 59.3)	23.2 (10.2, 36.1)	0.30
Moderate-to-vigorous physical activity	11.0 (1.3, 20.6)	11.2 (− 0.7, 23.1)	0.86	6.4 (− 3.5, 15.6)	19.6 (9.7, 29.4)	0.10	9.2 (− 0.5, 16.5)	11.7 (− 5.8, 29.2)	0.83

Models adjusted for all covariates except for the stratification variable. Non-current users (*n* = 1,779) were the reference group. The difference in means was calculated by subtracting non-current user estimates from current cannabis user estimates. All estimates are weighted due to the survey design. CI = Confidence interval. Bolded text represents statistically significant effects

**Table 4:** Adjusted odds of self-reported moderate-to-vigorous physical activity by cannabis use frequency

	Light users *n* = 144	*p*	Moderate users *n* = 50	*p*	Frequent users *n* = 55	*p*
	Reference group = non-current users (*n* = 1843) *OR* (95% CI)					
*Self-reported moderate-to-vigorous physical activity*						
Model 1	1.08 (1.01, 1.15)	**0.03**	0.94 (0.75, 1.18)	0.61	0.90 (0.75, 1.09)	0.31
Model 2	1.06 (0.99, 1.13)	0.14	0.93 (0.75, 1.14)	0.49	0.88 (0.73, 1.06)	0.20
Model 3	1.09 (1.03, 1.15)	**0.01**	0.98 (0.82, 1.16)	0.78	1.00 (0.87, 1.16)	0.98

Model 1: unadjusted model (*N* = 2,092); Model 2: adjusted for age and sex (*N* = 2,092); Model 3: adjusted for age, sex, race/ethnicity, education, income-to-poverty ratio, body mass index, cigarette smoker status, and alcohol use (*N* = 2,022). *OR* = Odds ratio; CI = confidence interval. Bolded text represents statistically significant effects

### Sensitivity analyses

Results did not substantially differ when excluding participants with diabetes, arthritis, heart failure, coronary heart disease, or cancer. However, upon excluding participants with less than four days of adherent accelerometer wear time, a few differences were observed. There were no longer significant differences between frequent cannabis users and non-current users in minutes engaged in light PA or MVPA (light PA: 386.2 min/day (95% CI [355.4, 417.1]) vs. 357.7 min/day (95% CI [347.2, 368.2]), *p* = 0.14; MVPA: 33.1 min/day (25.4, 40.8) vs. 27.7 min/day (26.3, 29.0), *p* = 0.21). Additionally, light cannabis use was a marginally significant predictor of self-reported engagement in MVPA (odds ratio [*OR*] = 1.07, 95% CI [1.00, 1.15], *p* = 0.08).

## Discussion

To our knowledge, this is the first study to use objective accelerometry measures to assess the relationship between cannabis use and physical behavior in a population-based sample of US adults. The results suggest that frequent cannabis users engaged in more PA than non-current users, but spent similar amounts of time in SB. Light and moderate cannabis users did not differ from non-current users in minutes spent in PA per day. Additionally, the associations between cannabis use and PA were stronger among adults over 40 years old and those who did not currently smoke cigarettes. Lastly, the subjective measures of MVPA produced different results, such that light cannabis users had greater odds of self-reporting MVPA compared to non-current users.

Our results can be compared to the analysis of 2007–2014 NHANES data by Vidot et al. [19]. Using self-reported PA assessments, they found a lower prevalence of engaging in MVPA among current cannabis users compared to non-current users, and found that as frequency of cannabis use increased, the minutes spent in MVPA decreased. We did not find the same pattern of results for accelerometer-measured or self-reported MVPA (with self-reported MVPA measured identically to their main outcome). Our results show that, in contrast, frequent cannabis users spent more minutes in MVPA and had similar odds of self-reporting MVPA compared to non-current users. Vidot et al. [19] examined data from a longer time period than the current study, yielding nearly 6.5 times as many cannabis users for analyses, which may contribute to the discrepancy between results. Furthermore, given the well-documented inconsistencies between objectively and subjectively reported PA, it is possible that self-report biases contribute to the Vidot et al. [19] findings. Our self-reported MVPA measure did not reveal any differences between frequent cannabis users and non-current users, indicating that objective versus subjective measures lead to differences when assessing the relationship between cannabis use and physical behavior.

Our findings do not support the mainstream perception of cannabis users as living sedentary lifestyles [18]. As cannabis’s legal status and risk perception changes [10], cannabis users have sought to challenge this stereotype, as demonstrated by the annual 420 Games, which feature a 4.2-mile run and other athletic competitions [41]. While the relationship between cannabis use and physical behavior has yet to be definitively established, it is possible that public perception of cannabis users has shifted, especially after the federal legalization of Cannabidiol (CBD) in 2018. This prospect should be explored in future research.

Age was negatively correlated with MVPA and positively correlated with SB in our sample, which is expected since the aging process contributes physiological barriers to engaging in high intensity PA [42]. We observed a larger difference in minutes of MVPA among participants above the median age (40 years) compared to those below, based on current cannabis use status (Table 3). Though not conclusive, this suggests that moderate cannabis use may have greater implications for PA in middle age. If true, a possible explanation is that cannabis is being used for exercise-induced pain recovery, since PA brings about pain and muscle soreness [43], and a decreased pain threshold and muscle hypersensitivity have been documented with increasing age [44]. Future research should assess age differences in physical behavior by cannabis use status among a larger age range, including older adults.

Prior research has posited that the endocannabinoid system (i.e., cannabinoids occurring naturally in the body) is a pathway by which moderate-to-vigorous physical activity (MVPA) leads to a psychologically rewarding, PA-induced state (e.g., runner’s high), which may play a role in the motivation to be physically active. The endocannabinoid pathway may be disturbed by the consumption of exogenous cannabinoids (i.e., cannabis use), but this mechanism is not fully understood [45, 46]. Alternatively, it has been suggested that exogenous cannabinoids may aid in recovery from pain and muscle soreness brought about by PA [43], thereby positively influencing the motivation to engage in PA [46]. Cannabis use has been shown to reduce pain and inflammation among patient populations [47–49]. However, among the general population, the evidence for an inverse association between cannabis use and inflammation is limited [31, 50].

Whereas frequent cannabis use predicted more minutes spent in light PA and MVPA, light cannabis use did not. This could potentially be due to differences in the reasons for cannabis use among frequent versus light cannabis users. Perhaps frequent users in the sample used cannabis for PA-related purposes and incorporated it into their lifestyle. Among light users, cannabis may have primarily been used recreationally. Participants did not report their reasons for cannabis use in NHANES, so future research is needed to examine how this may impact the relationship between cannabis use and physical behavior.

An important limitation to this study is the cross-sectional design, which does not allow the causality between cannabis use and levels of physical behavior to be determined. Second, accelerometer data were only available for the 2005–2006 NHANES. Although the legal and social context surrounding cannabis use have changed since the data used in this study were generated, this work establishes the need to incorporate objective measures into future studies and the results serve as a baseline with which future work can be compared. Having only one wave of NHANES data also led to small samples sizes when stratifying adults by cannabis use status, and future studies with larger samples are needed to improve precision. Third, the NHANES Drug Use Questionnaire did not distinguish between strains of cannabis, which have varying levels of tetrahydrocannabinol, the psychoactive component in cannabis, and are known to produce differing experiences for users [51, 52]. These differences may lead to distinct relationships with physical behavior. Lastly, accelerometers were not waterproof and thus were unable to capture any water-based PA, such as swimming.

Despite its limitations, this study was novel in its use of objective accelerometer data within a national, population-based survey. The objective accelerometry measures used in this study allowed physical behavior to be described with increased accuracy. Future studies using this approach should investigate more nuanced features of the cannabis–physical behavior relationship, including the precise timing and duration of SB and PA events.

## Conclusions

In a national, population-based US sample, current cannabis use was significantly associated with accelerometer-measured PA, such that frequent cannabis users engaged in greater minutes of light PA and MVPA compared to non-current users. Current and non-current cannabis users did not differ in time spent in SB. Results derived from self-reported MVPA did not align with those produced by accelerometer measures. Findings tended to be stronger among adults over 40 and those who did not smoke cigarettes. Findings add to the cannabis and physical behavior literature by incorporating objective accelerometer measures. Further understanding of the association between cannabis use and health behaviors is essential to fully addressing the public health concerns associated with cannabis use.

## Data Availability

The datasets used and/or analyzed during the current study are available in the Centers for Disease Control and Prevention/National Center for Health Statistics repository. https://wwwn.cdc.gov/nchs/nhanes/Default.aspx

## Abbreviations

PA: Physical activity
SB: Sedentary behavior
NHANES: National Health and Nutrition Examination Survey
MVPA: Moderate-to-vigorous physical activity
cpm: Counts per minute
BMI: Body mass index
SD: Standard deviation
GED: General education development
AA: Associates of arts
